# Effect of Transglutaminase Pre-Crosslinking Treatment Incorporated with Glucono-δ-lactone on the Physicochemical and Digestive Properties of Tofu

**DOI:** 10.3390/polym14122364

**Published:** 2022-06-11

**Authors:** Tianran Hui, Guangliang Xing

**Affiliations:** 1 School of Biology and Food Engineering, Changshu Institute of Technology, Changshu 215500, China; thui@troy.edu; 2 Department of Biological and Environmental Sciences, Troy University, Troy, AL 36082, USA

**Keywords:** tofu, transglutaminase, glucono-δ-lactone, in vitro digestion, bioaccessibility

## Abstract

This study evaluated the effect of transglutaminase (TGase) pre-crosslinking treatment on the physicochemical and digestive characteristics of tofu coagulated by glucono-δ-lactone (GDL). Results showed that certain TGase pre-crosslinking times (0.5, 1, 2 and 3 h) could promote the colloidal stability of soymilk with increased particle average sizes and absolute values of zeta potential. Particularly, the water holding capacity and gel strength of tofu pre-crosslinked by TGase for 2 h were 6.8% and 47.7% enhancement, respectively, compared to the control, and exhibited the highest score of overall acceptability. However, extensive pre-crosslinking by TGase for 3 h had an adverse impact on the sensory of tofu with poor firmness, rough structure and whey separation. Hence, the tofu gel pre-crosslinked by TGase for 2 h and then coagulated by GDL was recommended which showed a “slow release” mode of soluble proteins during the in vitro digestion phase, and had more chances to release bioactive peptides than soymilk.

## 1. Introduction

As a high quality and rich source of protein, the protein content of soybean (*Glycine max*) varies according to different varieties as 30~40% (*w*/*w*). Soybean curd, also named as tofu, is made from heated soymilk with added coagulants [1]. The yield and quality of tofu are largely determined by the type of coagulant used. Traditionally, the common coagulants could be divided into three categories, namely, salts (such as MgCl_2_ and CaSO_4_), acids (such as lactic acid and glucono-δ-lactone, GDL) and enzymes (such as chymosin and transglutaminase, TGase, EC 2.3.2.13) [2].

Nevertheless, each kind of coagulant has shortcomings in the tofu production process, for example, a rapid release of MgCl_2_ or residual undissolved CaSO_4_ could result in a rough texture, poor water holding capacity (WHC) and unpleasant taste of the final product [3,4]. Tofu made by GDL has a soft, tender texture and is not suitable for frying. The gel strength of tofu made with TGase is generally low, and the production process is often time-consuming [5]. As mentioned above, a single coagulant has disadvantages, while mixed coagulants are used with the development of automation, mechanization and industrialization, which can give the product a better texture and taste. Zhao et al. [6] investigated that the addition of salt-type coagulants could improve the mechanical properties of citric acid-induced tofu. Wang et al. [7] reported that a kind of fermented tofu, prepared by combining salt coagulants with lactic acid bacteria, increased hardness, yield and obtained a dense structure. Although the physicochemical characteristics of tofu induced by mixed coagulants (e.g., yield, WHC, gel firmness, rheological property, etc.) were often studied, few research was forced on the nutritional properties. It was reported that different coagulants could influence the protein bioaccessibility of soy protein gels [8]. “Bioaccessibility” means that the ingested nutrients are absorbed and utilized by the host organism [9].

Therefore, this work was aimed to evaluate TGase pre-crosslinking treatment incorporated with GDL on the physicochemical characteristics and protein bioaccessibility of tofu. In the present study, texture profile analysis, WHC and sensory analysis were carried out to assess the strength, rigidity and acceptability of tofu obtained by treating soymilk with TGase for different incubation times (0, 0.5, 1.0, 2.0 and 3.0 h) and then coagulated by GDL. Protein degradation profiles, soluble protein content and peptide content were investigated to evaluate the protein digestibility of tofu through an in vitro gastrointestinal model. It will contribute to understanding the use of mixed coagulants (TGase combined with GDL) in the tofu manufacturing industry.

## 2. Materials and Methods

### 2.1. Materials

Soybeans were purchased from a market in Changshu, China. TGase (100 U/g) was bought from Yiming Biological Technology Co., Ltd., Taixing Jiangsu, China. GDL was obtained from Xinhuanghai Food Co., Ltd., Zhangshu Jiangxi, China. α-Amylase (G8290), pepsin (P8390), pancreatin (P7340) and bile acid (G8310) were obtained from Solarbio Science and Technology Co., Ltd., Beijing, China.

### 2.2. Soymilk and Tofu Preparation

The selected soybeans (200 g) were soaked with 3-fold distilled water overnight. After soaking, 1400 mL of distilled water were added and the soybeans were ground by a food blender (L18-Y915S, Joyoung Co., Ltd., Hangzhou, China). Then, a 160-mesh screen was used to remove okara. The raw soymilk was collected and heated to 100 °C for 5 min with regular stirring. The cooked soymilk was cooled to ~50 °C and then divided into five equal volume parts. Each part was pre-crosslinked by TGase (3.0 U/g protein) at 50 °C for different time spans (0, 0.5, 1, 2 and 3 h). Subsequently, each part was mixed with 0.3% (*w*/*v*) GDL and then heated at 85 °C for 30 min to make tofu gels.

### 2.3. Particle Size and Zeta Potential Measurement

The soymilk samples pre-treated by TGase for different time spans as mentioned in Section 2.2 were diluted by distilled water to 1.0 mg protein/mL. The particle size and zeta potential of the diluted samples were determined by Zetasizer Nano (ZS90, Malvern Instruments Ltd., Malvern, UK), according to a report by Xing et al. [10].

### 2.4. Determination of Gelation Properties

The WHC of tofu samples (pre-crosslinked by TGase for different time spans and then coagulated by GDL) as mentioned in Section 2.2 were measured. The tofu gels were cut into columned shapes (2.0 cm diameter × 1.0 cm height) and then centrifuged at 480× *g* for 10 min as the method adapted from Peng and Guo [11].

Texture profile analysis (TPA) was performed to evaluate the gel strength of tofu by using a TA.X2i texture analyzer (Godalming, Surrey, UK), equipped with a TA50 probe (diameter: 50.0 mm), according to a previous report [12].

### 2.5. Sensory Evaluation

The sensory characteristics of tofu (pre-crosslinked by TGase for different time spans and then coagulated by GDL) were evaluated by 10 trained panelists. Color, overall acceptability, firmness, mouthfeel and flavor were selected as indexes for indicating the tofu quality. A 5-point scale was used to record the results for each attribute according to Li et al. [4]. Water was available for members to cleanse their palates between every sample.

### 2.6. In vitro digestion

Four kinds of samples were subjected to in vitro gastrointestinal simulated digestion (GIS), i.e., (1) soymilk incubated with TGase at 50 °C for 2 h (ST), (2) soymilk incubated with GDL at 85 °C for 30 min (SG), (3) soymilk pre-treated by TGase at 50 °C for 2 h and then coagulated by GDL at 85 °C for 30 min (STG) and (4) the cooked soymilk without addition of TGase or GDL was set as control.

The in vitro GIS was performed in three sequential phases: buccal (P1), gastric (P2) and intestinal (P3) digestion, as previously described by Xing et al. [13] with slight modifications. Firstly, a hand-held homogenizer was used to grind the tofu gel 10 times to simulate the chewing process, and a 5 g undigested sample (P0) was collected. Subsequently, a 60 g ground sample was added to 24 mL of α-amylase solution (*w*/*v*, 0.2 mg/mL, 20 mM phosphate buffer, pH 7.0), the mixture was shaken in a 37 °C water bath at 55 r/min for 3 min to simulate buccal digestion and then 7 g of the sample was taken out as the buccal-digested sample (P1). The pH of the system was then adjusted to 2.0 with 4 mol/L HCl, 33 mL of pepsin solution (*w*/*v*, 3.2 mg pepsin/mL, 0.1 M HCl) was added, the speed was kept at 55 r/min and the gastric digestion time was 1 h. During this period, 10 g of digested samples were taken at 5 min and 60 min (P2-5, P2-60). After gastric digestion, the pH of the system was adjusted to 7.0 with 4 mol/L NaOH, we added 18 mL pancreatic juice and 18 mL of bile (*m*/*v*, 0.4 mg/mL, 20 mM phosphate buffer, pH 7.0) and the digestion was continued at 37 °C for 2 h at 150 r/min. During the intestinal digestion, 14 g samples were taken at 30 min and 120 min (P3-30, P2-120). The digested samples collected at each digestion stage were filled up with distilled water to 14 mL to ensure that the protein content in all samples was consistent, and then were placed in a water bath at 90 °C for 5 min to inactivate the enzyme. All the digested samples were centrifuged at 8000× *g* for 15 min and the supernatants were collected to store at 4 °C until use.

### 2.7. Sodium Dodecyl Sulfate-Polyacrylamide Gel Electrophoresis (SDS-PAGE)

The samples as mentioned in Section 2.2 and progressive protein degradation during GIS as mentioned in Section 2.6 were analyzed by SDS-PAGE with 4% (*w/v*) stacking gel and 12% (*w*/*v*) separating gel. Twenty μL sample was mixed with an equal volume of buffer (0.1 M Tris buffer, 2% SDS, 10% β-mercaptoethanol, 20% glycerol, pH 6.8) and heated for 5 min in boiling water. Then, 20 μL of each sample was applied to each line. After electrophoresis, gels were stained with Coomassie brilliant blue R250 (0.1%, *w*/*v*) in a solution containing 42% ethanol and 10% acetic acid for 3 h and were destained with 45% ethanol and 5% acetic acid. Molecular mass standard (15, 20, 25, 35, 50, 70, 100, 130 kDa, Biosharp, Labgic Technology Co., Ltd., Hefei, China) was used. The SDS-PAGE gels were analyzed by Quantity One software (Version 4.6.2, Bio-Rad Laboratories, Inc., Hercules, CA, USA).

### 2.8. Total Soluble Protein Content Measurement

The soluble protein content of digested samples collected at each digestion stage mentioned above was measured according to the Bradford assay [14]. The concentration was measured at 595 nm by an ultraviolet-visible spectrophotometer (UV-1800, Mapada Instruments Co., Ltd., Shanghai, China). Bovine serum albumin was used as a standard.

### 2.9. Peptide Content Measurement

The digested samples taken at P0, P1, P2-60 and P3-120 were passed through ultrafiltration membranes (Millipore, Bedford, MA, USA) with molecular weight (MW) cut-offs of 10 kDa. This penetrating fluid was defined as small peptides (MW < 10 kDa). Fifty μL filtrate was added to a pre-prepared 2 mL reagent as previously described by Chen et al. [15]. Casein tryptone was used as a standard.

### 2.10. Statistical Analysis

All experiments were performed at least three times, data are expressed as the means ± standard deviation of three replicates. The SPSS software (Version 16.0, SPSS Inc., Chicago, IL, USA) was used to analyze the statistical results. Analysis of variance (ANOVA) and Duncan’s multiple comparison tests were used to determine the significant differences between means, and *p* < 0.05 was considered as significant.

## 3. Results and Discussion

### 3.1. Particle Average Size and Zeta Potential Analysis

The effects of incubation time (0, 0.5, 1, 2 and 3 h) on the particle average size and zeta potential of TGase-crosslinked soymilk at 50 °C are shown in Table 1. As time went on, larger and larger protein aggregates were formed; the cross-linking reaction caused by TGase was responsible for this result. It is well known that TGase is able to catalyze intra- and/or intermolecular crosslinks among protein molecules that result in forming ε-(γ-glutamine) lysine covalent isopeptide bonds [16]. As seen from Table 1, even if the incubation time lasts for only 0.5 h, significant increase (*p* < 0.05) in the particle average size (0.5 h, 442.2 ± 5.1 nm) was observed compared to the soymilk before incubation with TGase (0 h, 362.9 ± 3.2 nm). Moreover, TGase treatment also caused a gradual promotion in the absolute value of zeta potential with increasing time. The zeta potentials for TGase-treated soymilk samples were highly negative, from −26.5 ± 0.7 mV (0 h) to −38.9 ± 3.4 mV (3 h), while the pH values had no significant changes during the incubation period (6.72 (0 h) to 6.77 (3 h), data not shown). This observation was in accordance with Fu and Zhao [17], who revealed that soy protein isolate emulsions treated by TGase for 3 h had increased zeta potentials from −27.7 mV to −30.7 mV and hydrodynamic radius from 82.9 nm to 101.0 nm. It reported that the absolute zeta potential values for proteins greater than 20 mV could provide sufficient repulsion and ensure good dispersion stability [18]. Hence, TGase pre-crosslinking treatment promoted the colloidal stability of soymilk samples.

### 3.2. Electrophoresis of Soymilk Samples Treated by TGase Combined with GDL or Not

**Figure 1:**
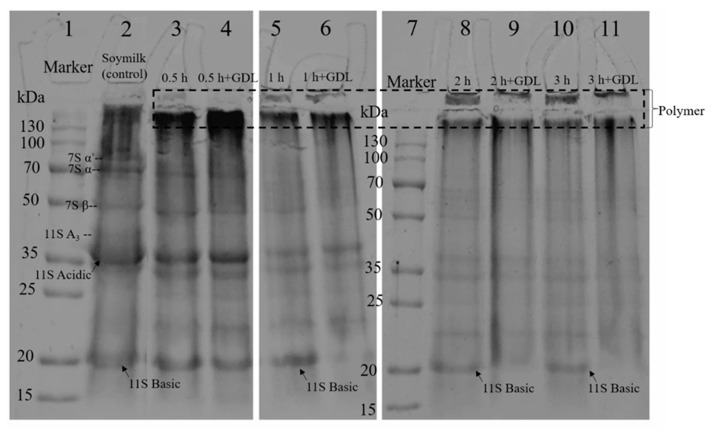
SDS-PAGE profiles of soymilk samples pre-incubated with TGase for various periods of 0.5, 1, 2 and 3 h, respectively, (corresponding to lanes 3, 5, 8 and 10, respectively) and then coagulated by GDL (corresponding to lanes 4, 6, 9 and 11, respectively).

The effects of TGase combined with GDL or not on protein crosslinking were visualized by SDS-PAGE; the major soy protein bands were clearly separated as shown in Figure 1. β-Conglycinin (7S) is a trimeric glycoprotein composed of α’, α and β subunits. Glycinin (11S) is a hexameric protein consisting of acidic and basic subunits [19]. A smear in the high MW area (>130 kDa, framed in black line) was observed above the stacking gel in all samples, indicating that TGase-induced polymerization occurred. Meanwhile, a gradual decrease in the quantity of 7S and 11S proteins was noticed as time went on.

In particular, with only TGase treatment for 0.5 h (lane 3, Figure 1), the 7S α’ and 11S A3 subunits disappeared. The 7S α’ and 11S A3 subunits might be located on the surface of 7S and 11S globulins, respectively, and thus, might be attacked by TGase easily [20]. A similar profile was obtained in the soymilk sample which was treated with TGase for 0.5 h firstly and then coagulated by GDL (lane 4, Figure 1). It is worth noting that the content of high-MW-crosslinked proteins was higher in lane 4 compared to lane 3, as evidenced by the increased intensity of protein staining at the top of the gel. Therefore, soymilk pre-crosslinked by TGase and then coagulated by GDL was helpful to promote the polymerized reactions among proteins. This view was supported by the other three groups of soymilk samples which were treated by TGase for 1, 2 and 3 h firstly and then incubated with GDL (lanes 5~11). As can be seen clearly from lane 6, the 11S basic subunits disappeared compared to lane 5, as well as in lane 8 and lane 10. Most likely, the combined use of TGase and GDL showed enhanced capacity to crosslink 11S basic subunits, and much larger aggregates formed which were hard to penetrate the stacking gel. Accordingly, our results suggested that TGase in conjunction with GDL treatment might accelerate soybean protein polymerization.

### 3.3. The WHC and Gel Strength

**Figure 2:**
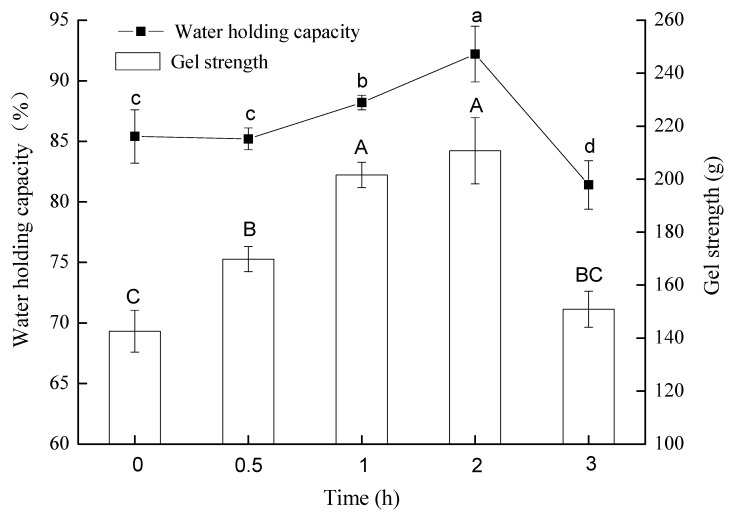
Changes in water holding capacity and gel strength of tofu prepared from soymilk with different TGase pre-crosslinking time spans (0, 0.5, 1, 2 and 3 h) and then coagulated by GDL. Means in the same plot that do not share a common superscript letter are significantly different (*p* < 0.05).

The consumption qualities of tofu are usually evaluated by the structural rigidity of protein networks and the uniformity of gel textures. In fact, these gel properties could be illustrated by gel strength (obtained through texture profile analysis) and WHC (obtained through centrifugation) [21]. The effect of pre-crosslinking time by TGase on the gel strength and WHC of tofu are presented in Figure 2. When the TGase incubation time was increased from 0 h to 2 h, the gel strength of tofu increased by about 47.7% from 142.6 ± 7.9 g to 210.6 ± 4.8 g and then reduced to 150.88 ± 6.8 g after 3 h of TGase pre-treatment. Similarly, the WHC of tofu was increased from 85.4% ± 2.2% to 92.2% ± 2.3% during the first 2 h with TGase pre-treatment and then reduced dramatically to 81.4% ± 2.0% after 3 h of TGase pre-treatment. It has been reported that the primary factors to determine the WHC of gel are structure and strength, uniform and strong structures inclined to “entrap” water within the protein networks [22]. In this research, it can be speculated that TGase pre-treatment (from 0 to 2 h) caused strong crosslinking of soy proteins (indicated by the increased particle size of protein aggregates as shown in Table 1); the finally GDL-induced tofu had a denser protein structure, which potentially helped to increase the gel strength and WHC. However, excessive crosslinking (pre-treated by TGase for 3 h) inhibited the uniform development of the protein network and had negative effects on the gel properties. A similar conclusion was reached by Luo et al. [23], who reported that excessive TGase crosslinking of soy protein isolate led to the formation of too large polymers or aggregates that were hard to form an effective network.

### 3.4. Sensory Evaluation

**Figure 3:**
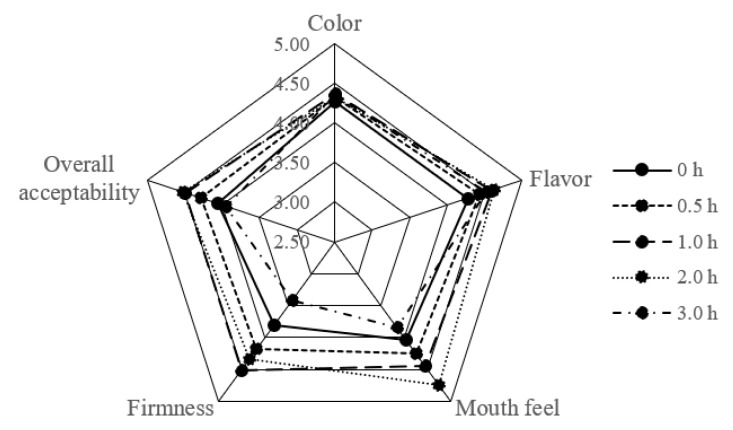
Sensory evaluation of tofu prepared from soymilk with different TGase pre-crosslinking times (0, 0.5, 1, 2 and 3 h) and then coagulated by GDL.

The sensory properties of tofu samples prepared from soymilk with different TGase pre-crosslinking time spans (0, 0.5, 1, 2 and 3 h) and then coagulated by GDL were evaluated; the results are shown in Figure 3. It indicated that TGase pre-crosslinking treatments from 0 h to 2 h had a positive effect on improving consumer acceptance. Particularly, in terms of texture, the firmness scores improved with increasing the pre-crosslinking time of TGase from 0 h to 2 h. These results were in compliance with the gel strength results, which suggested that the tofu texture became uniform and denser (Figure 2 and Figure 3). By implementing pre-crosslinking treatment (from 0 h to 2 h), the mouthfeel of tofu could be improved. However, when the pre-crosslinked time by TGase was 3 h, both firmness and mouthfeel of tofu were significantly decreased; excessive TGase crosslinking might be responsible for this result, as mentioned in Section 3.3. Color difference and flavor were similar among tofu groups. Hence, too long pre-crosslinking time could impair the sensory perception of tofu. In general, the tofu with a 2 h pre-crosslinking period by TGase treatment and then coagulated by GDL got the highest scores of overall acceptability which consisted with the results of WHC and gel strength analysis; it was selected for the following digestive experiment.

### 3.5. Protein Degradation

**Figure 4:**
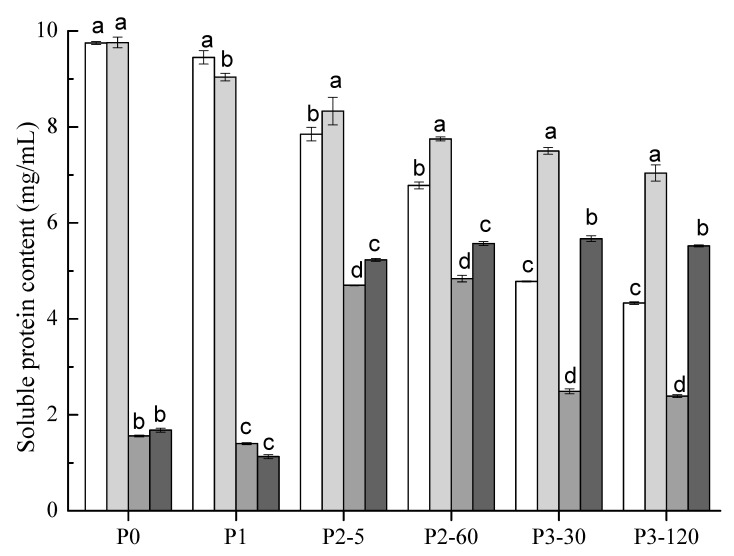
Soluble protein content (mg/mL) of soymilk (white), ST (light gray), SG (gray) and STG (black) samples at different phases of in vitro GIS. Within the same digestive phase, different lowercase letters indicate a significant difference (*p* < 0.05).

The changes of soluble protein contents in soymilk (the control), ST, SG and STG samples during gastrointestinal phases are shown in Figure 4. ST had no dramatic difference (*p* > 0.05) compared to the control but showed a significantly higher (*p* < 0.05) soluble protein content of 9.76 mg/mL than that of SG and STG (1.56 mg/mL and 1.68 mg/mL, respectively) before digestion (P0 stage). The different gelation mechanisms of GDL and TGase were responsible for this phenomenon. For SG, the protons released by the addition of GDL neutralized the surface negative charges of soluble aggregates in heated soymilk. This led to a reduction in electrostatic repulsion, and thus promoted the formation of van der Waals attraction and the hydrophobic force among proteins and then induced the coagulation [24]. In contrast, with regard to ST, the negative charges could not be neutralized and covalent bonds were formed among soy proteins by TGase-catalyzed crosslinking. According to our previous study, only added TGase (3.0 U/g protein) to soymilk was impossible to induce gelation even after a 4 h incubation period [25], hence ST presented as a liquid state. It is within expectation that SG and STG gels had a much lower protein solubility than that of ST before digestion (P0). Similar results were observed after buccal digestion (P1), which suggested that the α-amylase in simulated saliva solution had few effects on the soluble protein content.

The SG had a similar soluble protein content changing pattern compared to STG during gastric digestion (P2) stages which maintained a higher level compared to that of P0 and P1 phases. It was largely due to the enzymatic hydrolysis of pepsin. In terms of the P2 stage, STG had significantly higher (*p* < 0.05) soluble protein contents of 5.23~5.57 mg/mL than SG of 4.72~4.85 mg/mL. However, during the latter P3 stage, the solubility of proteins in STG were maintained high (5.52~5.67 mg/mL), but in SG were reduced significantly (2.39~2.49 mg/mL). It seems that the soluble proteins in STG showed a “slow release” mode during the whole digestion phase. It is worth noting that both the control and ST showed lower soluble protein content during intestinal digestion (P3) phases compared to the gastric digestion (P2) phases. It might be due to that many large proteins were hydrolyzed by pepsin and/or pancreatin to form peptides with lower molecular weights and amino acids, which were hard to detect by Bradford assay [8].

### 3.6. Bioaccessible Peptides

**Figure 5:**
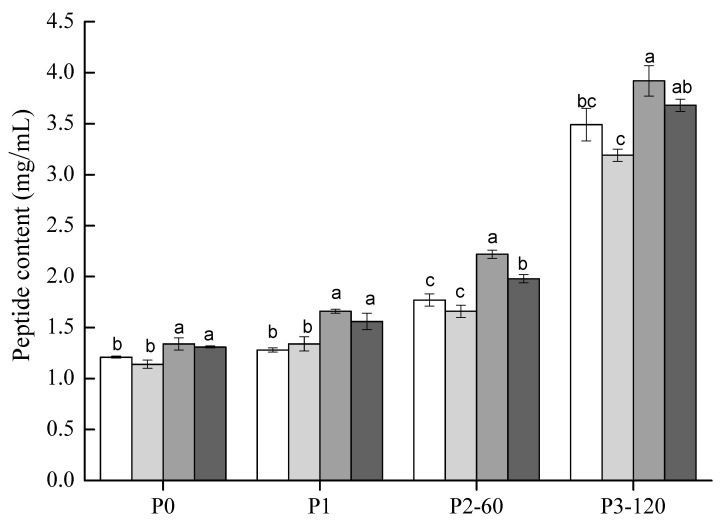
Peptide contents (<10 kDa) of soymilk (white), ST (light gray), SG (gray) and STG (black) samples at different phases of in vitro GIS. Within the same digestive phase, different lowercase letters indicate a significant difference (*p* < 0.05).

The peptide (<10 kDa) contents of all the investigated samples are shown in Figure 5. For all samples, before digestion (P0), the amounts of peptides remained very low (1.14~1.34 mg/mL), as well as after the oral digestion phase (P1). It indicated that neither chewing nor salivary amylase could hydrolyze soy proteins. Both SG and STG showed significantly higher (*p* < 0.05) levels of peptide contents than the control and ST. As the GIS digestion progressed, the peptide contents of all samples increased gradually; the digestive enzymes in gastric and duodenal juices contributed to these results. At the end of gastric digestion (P2-60) and intestinal digestion (P3-120), SG maintained the highest peptide contents among the tested samples. Although no remarkable difference (*p* > 0.05) was obtained between SG and STG at P3-120, a higher value (*p* < 0.05) was observed than the control and ST. It was reported that small molecular weight peptides (<10 kDa) from legume proteins were mostly with bioactivities [26]. Our results suggested that, during in vitro GIS digestion, SG and STG samples had more chances to release bioactive peptides than soymilk and ST.

### 3.7. SDS-PAGE Profiles of Digesta

**Figure 6:**
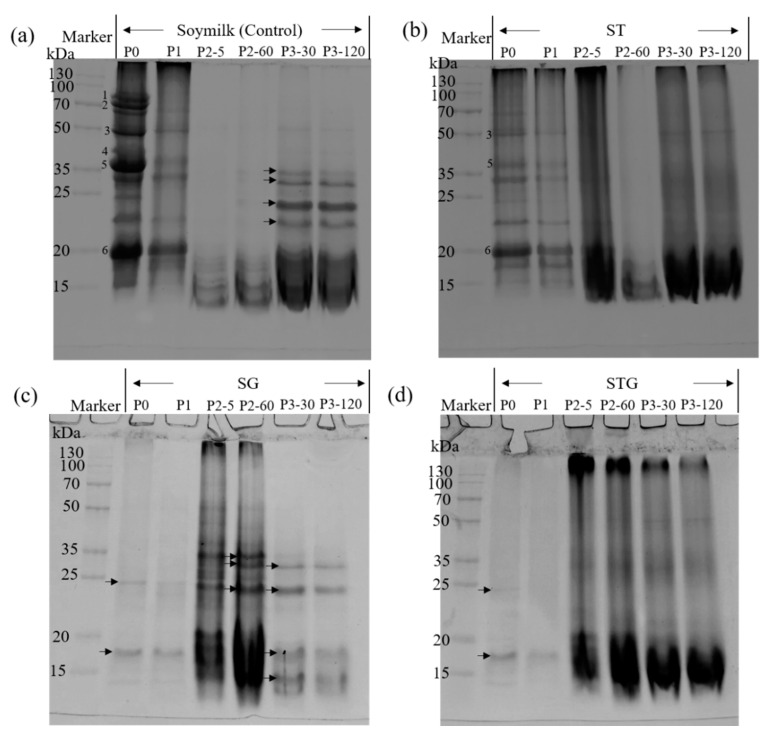
SDS-PAGE profiles of proteins collected from supernatant at different phases of in vitro GIS. P0 represents sample before the GIS digestion; P1 represents sample after buccal digestion; P2-5, P2-60 represent samples taken at 5 min and 60 min of gastric digestion; P3-30, P3-120 represent samples taken at 30 min and 120 min of intestinal digestion. (**a**) soymilk, (**b**) soymilk incubated with TGase (ST), (**c**) soymilk incubated with GDL (SG), (**d**) soymilk pre-treated by TGase and then coagulated by GDL (STG).

The protein profiles of each sample during gastrointestinal phases were visualized by SDS-PAGE (Figure 6). As shown in Figure 6a, before digestion (lane P0), the main components of soy protein in the soymilk sample (the control) were clearly observed, which were speculated to be 7S α′, 7S α and 7S β subunits (corresponding to band number 1, 2 and 3, respectively), 11S A3 subunit (band number 4), 11S acidic subunits (band number 5) and 11S basic subunits (band number 6) based on their molecular masses of 75.2, 66.6, 47.7, 41.1, 35.3 and 19.7 kDa [27]. It could be clearly seen that 7S α′ and 7S α were absent in the soluble fractions of ST; 11S A3 subunit was also depleted, followed by the 11S acidic subunits (band 5) and 11S basic subunits (band 6) (lane P0, Figure 6b). Meanwhile, high MW polymers (>130 kDa) were obtained at the top of the gel which suggested that protein polymerization did occur in the presence of TGase. The enzymatic crosslinking reactivity for the acidic subunits of 11S was higher than that for 11S basic proteins, which is inconsistent with Hsieh et al. [20]. Both SG and STG gels showed less bands at P0 and P1 stages than soymilk, and only two major bands (indicated by the arrows in Figure 6c,d, with a corresponding molecular mass of 24.5 and 17.4 kDa) could be observed, suggesting that most proteins were trapped in the soymilk gel. These results were consistent with the low soluble protein contents initially obtained in Figure 4.

Subsequent gastric digestion led to the loss of 7S and 11S globulins in all samples, but at the same time some smeared bands (15~35 kDa) appeared (P2-5 and P2-60, Figure 6). It indicated that a majority of the protein macromolecules underwent rapid degradation. During this phase, the acidic environment, the continuous mechanical shock, the presence of pepsin and the diluting effect of digestive enzyme solution led to the collapse of the organized protein network, thus promoting the release of soluble proteins. Other authors had reported similar observations [28,29]. After gastric digestion (P2-60), some blurry bands (<15 kDa) were observed in soymilk (Figure 6a). Three protein bands with a molecular mass of 32.6, 28.9 and 23.8 kDa (as indicated by arrows in lane P2-60, Figure 6c) and a smear of bands corresponding to peptides <20 kDa were obtained in SG, which indicated a much slower digestion rate of SG compared to the control. ST and STG digesta showed a similar electrophoretic profile during the whole gastrointestinal digestion phase; no intact bands were obtained and most of the high MW polymers could be digested to 15~130 kDa polypeptides. These results were consistent with the findings of soluble protein and bioaccessible peptides contents. During the intestinal digestion phase, intensive bands at 32.8 kDa, 28.5 kDa, 23.8 kDa and 22.1 kDa were obtained in soymilk (as indicated by arrows in lane P3-30, Figure 6a). Under the hydrolysis of pancreatin, these four bands seemed to be hydrolyzed partially but still existent even after duodenal digestion (lane P3-120, Figure 6a). Additionally, similar digestion patterns were observed in SG with four intensive bands (28.5 kDa, 23.7 kDa, 17.5 kDa and 14.0 kDa, as indicated by arrows) in lanes P3-30 and P3-120 (Figure 6c).

## 4. Conclusions

TGase pre-treatment could change the physicochemical characteristics of tofu coagulated by GDL. Particularly, pre-treatment of soymilk with a short incubation time (0.5, 1 and 2 h) by TGase improved the gel strength, WHC and overall acceptability, whereas extensive pre-crosslinking by TGase for 3 h resulted in a big decrease in gel firmness and sensory scores. Hence, TGase pre-treatment for 2 h was recommended. Subsequently, the protein bioaccessibility of STG tofu (pre-crosslinked by TGase for 2 h and then coagulated by GDL) was studied by an in vitro GIS model. Results showed that the soluble proteins in STG presented a “slow release” mode during the whole digestion phase, and had more chances to release bioactive peptides than soymilk. These results are of great potential value for the combined use of TGase and GDL in the tofu manufacture industry.

## Figures and Tables

**Table 1:** Zeta potential and particle average size of soymilk samples incubated with TGase at 50 °C for different time spans.

Time	Zeta Potential (mV)	Average Size (d/nm)
0 h	−26.5 ± 0.7 ^a^	362.9 ± 3.2 ^d^
0.5 h	−33.7 ± 0.8 ^b^	442.2 ± 5.1 ^c^
1 h	−36.4 ± 1.7 ^b,c^	474.4 ± 26.1 ^c^
2 h	−37.9 ± 1.0 ^c^	547.3 ± 19.6 ^b^
3 h	−38.9 ± 3.4 ^c^	581.8 ± 12.0 ^a^

Different lowercase letters (a to d) within a column indicate one-way ANOVA of the mean values is significantly different (*p* < 0.05).

## Data Availability

Not applicable.
